# Distinct 2D *p*(2 × 2) Sn/Cu(111) Superstructure at Low Temperature: Experimental Characterization and DFT Calculations of Its Geometry and Electronic Structure

**DOI:** 10.3390/nano15211684

**Published:** 2025-11-06

**Authors:** Xihui Liang, Dah-An Luh, Cheng-Maw Cheng

**Affiliations:** 1 School of Arts and Sciences, Guangzhou Maritime University, Guangzhou 510725, China; 2 Department of Physics, National Central University, Taoyuan 320317, Taiwan; 3 National Synchrotron Radiation Research Center, Hsinchu 300092, Taiwan; makalu@nsrrc.org.tw

**Keywords:** Sn/Cu(111) superstructure, adsorption site, density functional theory, scanning tunneling microscopy, angle-resolved photoemission spectroscopy

## Abstract

Atomically precise control of metal adatoms on metal surfaces is critical for designing novel low-dimensional materials, and the Sn-Cu(111) system is of particular interest due to the potential of stanene in topological physics. However, conflicting reports on Sn-induced superstructures on Cu(111) highlight the need for clarifying their geometric and electronic properties at low temperatures. We employed scanning tunneling microscopy (STM), low-energy electron diffraction (LEED), angle-resolved photoemission spectroscopy (ARPES), and density functional theory (DFT) to investigate submonolayer (<0.25 ML) Sn adsorption on Cu(111) at 100 K. We confirmed a *p*(2 × 2) Sn/Cu(111) superstructure with one Sn atom per unit cell and found that Sn preferentially occupies three-fold *hcp* sites. ARPES measurements of the band structure—including a ~0.3 eV local gap between two specific bands at the ${\bar{\Gamma }}_{2}$ point in a metallic overall electronic structure—were in good agreement with the DFT results. Notably, the STM-observed *p*(2 × 2) morphology differs from the honeycomb-like or buckled stanene structures reported on Cu(111), which highlights the intricate interactions between adatoms and the substrate.

## 1. Introduction

Atomically precise manufacturing, as a future ultimate manufacturing technology that may create entirely new materials, involves controlling the arrangement of atoms on the surface of substances [1,2,3,4]. The deposition of one kind of metal atom on the surface of another metal, as a simple platform for studying the arrangement of atoms on the surface of substances, has been widely used to modify or create novel surface properties [5,6,7,8,9,10]. Among numerous bimetallic systems, the Sn-Cu system has drawn much attention because it possesses diverse properties, especially since two-dimensional (2D) stanene have been observed on Cu(111) [11,12,13,14], and stanene is considered highly promising due to its unique and enriched properties such as topological superconductivity, a near-room-temperature quantum anomalous Hall effect, giant magnetoresistance, and enhanced thermoelectricity [14,15,16,17,18,19,20,21,22,23,24,25,,26,27].

Previous studies have demonstrated that Sn atoms can be easily incorporated into the top atomic layer of the Cu(111) surface, forming a surface alloy at or above room temperature [28,29,30,31,32]. Liang et al. were the first to achieve the deposition of 2D Sn atoms on a Cu(111) substrate at low temperature, and they proposed a *p*(2 × 2) structure containing a single Sn atom per unit cell [11]. However, recently Deng et al. used STM to observe the epitaxial growth of stanene on Cu(111). They found that this stanene has an unusually ultra-flat zero-buckling geometry and a honeycomb structure, which can be viewed as a (2 × 2) superstructure with two non-equivalent Sn atoms in one unit cell [12,33]. Interestingly, this ultra-flat stanene exhibits topological band inversion, which may open up opportunities for exploring device applications and understanding the fundamentals of topological physics. In addition, the growth of ultra-flat stanene on Cu(111) also indicates that the interaction between the substrate and stanene plays an important role in stabilizing the ultra-flat 2D structure. The two types of 2D Sn-based structures have similar (2 × 2) superstructures but different Sn atom arrangements, which suggests that different phases can form in the Sn/Cu(111) system.

In this work, we investigated submonolayer (<0.25 ML) Sn adsorption on Cu(111) at low temperature and reproduced the *p*(2 × 2) superstructure with a single Sn atom per unit cell. This *p*(2 × 2) superstructure was characterized using scanning tunneling microscopy (STM), low-energy electron diffraction (LEED), and angle-resolved photoemission spectroscopy (ARPES). STM observations confirmed the *p*(2 × 2) superstructure with one Sn atom per unit cell. Furthermore, we found good agreement between the ARPES results and first-principles calculations.

## 2. Materials and Methods

ARPES measurements were performed at an end station attached to the ultra-high-resolution and high-flux U9 cylindrical-grating beamline (BL21B1) of the National Synchrotron Radiation Research Center in Taiwan. A Cu(111) single crystal (diameter: 1 cm) was mounted on a low-temperature manipulator. The sample temperature, which could be adjusted from 100 K to 1000 K, was measured using a type K thermocouple junction mounted beside the sample. The Cu(111) single crystal was cleaned through repeated cycles of Ar^+^-ion sputtering and annealing until a sharp *p*(1 × 1) LEED pattern and the Shockley surface state were obtained [34].

Tin was evaporated from a Knudsen cell while the sample temperature was maintained at 100 K. The same evaporation conditions were used throughout the experiment. The deposition rate was ~0.04 monolayer (ML)/min, which was calibrated using a quartz-crystal microbalance. Here, 1.0 ML is defined as 1.77 × 10^15^ atoms/cm^2^—the density required to form one complete Cu(111) surface layer. The relative error of the deposition rate was less than 15%. ARPES spectra were measured with photon energies of 37 eV and 41 eV. Energy distribution curves (EDC) were recorded using an electron energy analyzer (Scienta SES-200, Scienta Scientific, Uppsala, Sweden) with a collection angle of 7 degrees, and the angular resolution was approximately 0.125°. To acquire photoemission spectra over a wide range of k_||_ (the component of the photoelectron wave vector parallel to the surface), we combined data from several consecutive measurements via simple linear combination in the overlapping region, without further intensity normalization. The overall energy resolution was better than 30 meV.

STM experiments were conducted using a microscope (RHK UHV 300, RHK Technology, Troy, MI, USA) equipped with a back-filled ion gun for sample cleaning and a Sn doser; the base pressure of the system was less than 1 × 10^−10^ Torr. A Cu(111) single crystal, mounted on a transferable sample holder, was heated via radiation and electron bombardment and cooled using liquid nitrogen, allowing the sample temperature to be adjusted from 100 K to 1000 K. The sample temperature was measured using a type K thermocouple junction attached directly to the sample. The sample was cleaned through cycles of Ar-ion sputtering and annealing. STM tips were fabricated from electrochemically etched W wire (diameter: 0.25 mm). All images were acquired under sample biasing and constant tunneling current conditions.

Density functional theory (DFT) calculations were performed using the PWscf code from the Quantum Espresso package [35,36]. The Perdew–Burke–Ernzerhof (PBE) exchange-correlation functional within the general gradient approximation (GGA) was employed. To describe electron–core interactions, we employed projector augmented-wave (PAW) pseudopotentials, which were sourced from the PSlibrary (v.1.0.0)—a publicly accessible, rigorously validated pseudopotential library hosted on the official Quantum Espresso website. For spin–orbit coupling (SOC) calculations, relativistic PAW pseudopotentials were adopted to account for relativistic effects in both Sn (a heavy element) and Cu. This choice ensured consistency in the electronic structure description between SOC and non-SOC calculations when toggling SOC on or off. The kinetic energy cutoff for the plane-wave basis and the k-point sampling for Brillouin zone integration were determined through systematic convergence tests, ensuring total energy convergence within 1 meV/atom (Supplementary Tables S1 and S2). Based on these tests, the kinetic energy cutoff was set to 800 eV, with a corresponding charge density cutoff of 8000 eV. A 28 × 28 × 1 Monkhorst–Pack k-point grid was employed for numerical integration. A Gaussian smearing of 0.1 eV was applied to stabilize self-consistent field convergence in the metallic Sn/Cu(111) system, and this smearing width was verified to have a negligible impact on the reported adsorption energies and electronic band structures. The equilibrium lattice parameter of 3.62 Å was obtained from structural optimization of bulk copper. The Cu(111) surface was modeled as a periodic slab consisting of six atomic layers, with four Cu atoms in each layer. The bottom three layers were fixed during relaxation to replicate the bulk copper geometry, while the upper layers were allowed to relax freely. A vacuum layer of ~15 Å was added between adjacent slabs to avoid significant interactions between periodically repeated slabs. Convergence tests for both the vacuum layer thickness and the number of Cu layers confirmed that 15 Å and 6 layers, respectively, are sufficient for converging the adsorption energy and electronic structure (Supplementary Tables S3 and S4). Spin–orbit coupling (SOC) was included in the self-consistent calculations of the electronic structure. To evaluate the influence of van der Waals (vdW) interactions, test calculations were conducted using the DFT-D3(BJ) correction scheme [37] as implemented in Quantum ESPRESSO. However, as discussed in Section 3.4.6, the D3(BJ) correction introduced artifacts consistent with those reported in metallic systems [38,39], and did not alter the qualitative conclusions of this work. Therefore, the primary results and analysis presented are based on the PBE functional.

## 3. Results and Discussion

### 3.1. Structural of p(2 × 2) Sn/Cu(111) Superstructure

**Figure 1:**
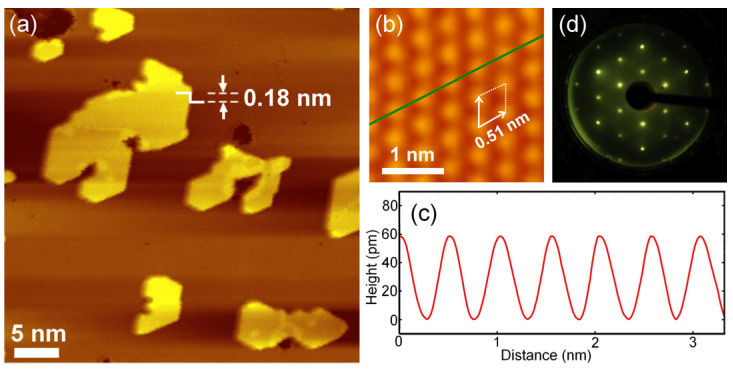
Atomic structures of the Sn/Cu(111) superstructure. (**a**) STM image of Sn deposited on Cu(111) with Sn coverage below 0.25 ML (sample bias *V_s_*  =  −0.50 V, tunnelling current *I*  =  0.25 nA). Sn coverage is controlled below 0.25 ML to avoid continuous film formation, resulting in isolated *p*(2 × 2) islands. (**b**) High-resolution STM image of the Sn/Cu(111) superstructure (*V_s_*  =  −0.50 V, *I*  =  0.28 nA). The bright spots correspond to individual Sn atoms, and the white dashed lines mark the boundaries of a *p*(2 × 2) unit cell. The experimental Sn-Sn distance (0.51 nm) is consistent with the DFT-calculated lattice constant of the *p*(2 × 2) Sn/Cu(111) supercell (0. 512 nm), confirming structural agreement between experiment and theoretical model. (**c**) Profile along the green line in (**b**) showing that the Sn atoms are identical in apparent height. (**d**) A LEED pattern of the Sn/Cu(111) superstructure.

Sn atoms were evaporated onto the Cu(111) surface (maintained at 100 K in ultrahigh vacuum), and the samples were characterized by LEED and STM without subsequent annealing. Figure 1a shows a representative STM topographic image of the sample with Sn coverage below 0.25 ML. The STM image reveals that parts of the Cu(111) substrate are covered by Sn islands, which clearly indicates a typical 2D growth mode. The apparent height of the Sn islands is 0.18 nm (Figure 1a), confirming that they are single atomic layers in thickness. The detailed atomic structure of a Sn island is visualized in Figure 1b, showing a lattice pattern with a lattice constant of 0.51 nm—exactly twice that of the Cu(111) substrate surface (2.55 Å). In the height profile (Figure 1c) along the green line in Figure 1b, all Sn atoms are clearly resolved and have identical heights, indicating that all Sn atoms occupy the same type of site on the Cu(111) surface.

**Figure 2:**
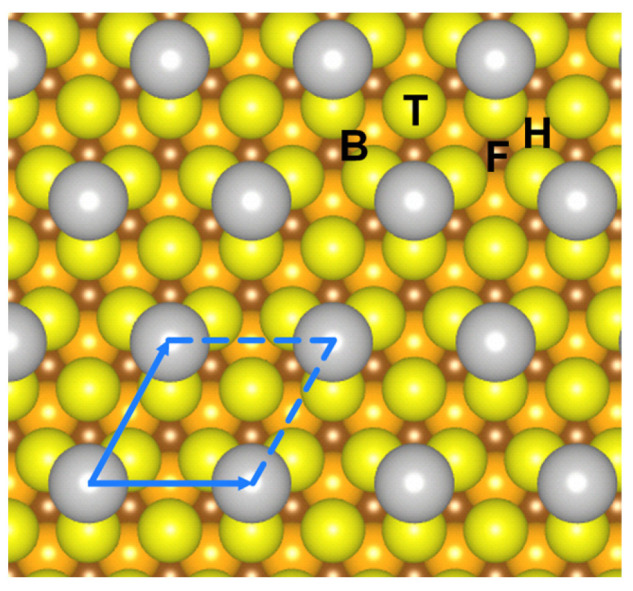
Schematic atomic model of the *p*(2 × 2) Sn/Cu(111) superstructure. Grey balls denote Sn atoms. Yellow, orange, and brown balls denote the Cu atoms in the topmost surface layer, the sub-surface layer, and the layer beneath the sub-surface of Cu(111), respectively. The blue parallelogram indicates one unit cell of the superstructure. Adsorption sites on the Cu(111) surface are labeled as follows: B (bridge site), T (top site), F (*fcc* site, with a Cu atom located two atomic layers below the surface), and H (*hcp* site, with a Cu atom located one atomic layer below the surface). The (2 × 2) supercell (blue parallelogram) matches the size used for adsorption energy calculations in Table 1, with Sn atoms (grey balls) occupying *hcp* sites in the ordered experimental structure.

Considering the corresponding LEED pattern (Figure 1d) and the relative orientation between the Sn islands and the substrate, the structure can be identified as a *p*(2 × 2) Sn/Cu(111) superstructure with one Sn atom per unit cell (Figure 2). This finding differs from honeycomb-like or buckled stanene, which contains two Sn atoms per unit cell [12,26,27,33].

### 3.2. DFT Calculation of Sn Adsorption Sites

To investigate the most stable adsorption sites of Sn on Cu(111) and their effects on the electronic structure, we calculated the adsorption energies of Sn atoms at different sites using DFT (see Table 1). Four types of sites were considered: top, 2-fold bridge, 3-fold *fcc*, and 3-fold *hcp* sites, denoted as T, B, F, and H, respectively, in Figure 2. Among these, the 3-fold *hcp* site is the most favorable, which is consistent with the results of previous DFT studies [40] and with the previously proposed structural model for the *p*(2 × 2) superstructure [11].

The *fcc* site is less stable than the *hcp* site, but the difference in adsorption energy between the two sites is only 0.01 eV—an observation directly tied to the high symmetry of the Cu(111) surface, a close-packed (111) face of face-centered cubic (*fcc*) metals. As visualized in Figure 2, both *fcc* and *hcp* sites are 3-fold hollow configurations surrounded by three nearest-neighbor Cu atoms in the top surface layer; their sole structural distinction lies in the position of Cu atoms in the subsurface layer: the *fcc* site is aligned above a Cu atom located two atomic layers below the surface, while the *hcp* site lies above a Cu atom located one atomic layers below the surface. This minimal structural difference leads to nearly identical Sn-Cu bonding environments—Sn’s 5*s*/5*p* valence orbitals interact equivalently with the three adjacent surface Cu atoms at both sites—resulting in the tiny 0.01 eV energy offset. This trend is consistent with literature: Yu et al. also reported a similarly small energy difference (<0.03 eV) between the *fcc* and *hcp* sites for Sn on a (3 × 3) Cu(111) surface, calculated using the local density approximation (LDA) with ultrasoft pseudopotentials (USPP) in the DMOL3 code [40]. Meier et al. reported a similar 0.01 eV energy difference between *fcc* and *hcp* sites for Sn on Au(111) [41].

To confirm that the optimized structures retain their initial adsorption sites, we performed two sets of relaxation calculations: (1) constrained relaxation (only vertical displacement of Sn atoms and the top three layers of the Cu(111) slab allowed, horizontal coordinates fixed) to isolate site-specific stability, and (2) unconstrained relaxation (both vertical and horizontal displacement permitted) to test for spontaneous site hopping. In constrained relaxation—used to generate the data in Table 1—all Sn atoms remained at their initial sites post-optimization. In unconstrained relaxation, Sn remained at the initial *fcc* or *hcp* site, with only vertical adjustments (±0.01 Å in Sn-substrate distance). This confirms that the optimized structures retain the initial adsorption sites, as the lateral energy barriers between adjacent sites (e.g., *hcp* to *fcc*) are sufficiently high to prevent migration during relaxation.

### 3.3. ARPES Analysis of Electronic Band Structure

**Figure 3:**
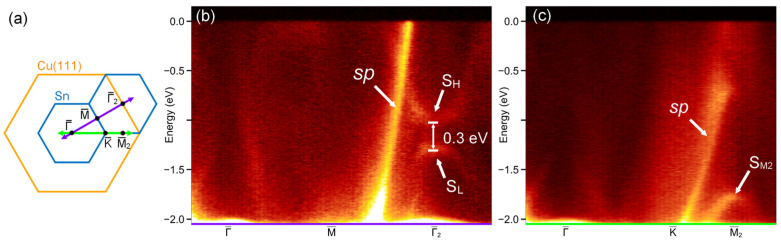
(**a**) 2D BZs of the *p*(2 × 2) Sn/Cu(111) superstructure. The second BZ is also shown. (**b**) ARPES spectra of the *p*(2 × 2) Sn/Cu(111) structure along the $\bar{\Gamma }-\bar{M}-{\bar{\Gamma }}_{2}$ (purple line in (**a**)) and (**c**) $\bar{\Gamma }-\bar{K}-{\bar{M}}_{2}$ (green line in (**a**)) directions. Energy is referenced to the Fermi level (*E_F_* = 0).

The discrepancy between our STM results and the reported honeycomb-like or buckled stanene on Cu(111) motivated us to further investigate the electronic structure of the *p*(2 × 2) superstructure. The surface Brillouin zones (BZs) of the *p*(2 × 2) structure are shown in Figure 3a. Figure 3b presents ARPES spectra of the *p*(2 × 2) structure along the $\bar{\Gamma }-\bar{M}-{\bar{\Gamma }}_{2}$ direction. Two dominant features can be identified in the right half of Figure 3b: the intensely bright Cu(111) substrate *sp* band that extends from a higher binding energy towards Fermi level (labeled as *sp* in Figure 3b) and a gap between two intense bands (labeled as S_H_ and S_L_ in Figure 3b). The gap between the two intense bands is located at the ${\bar{\Gamma }}_{2}$ point in the second BZ, which is equivalent to the $\bar{\Gamma }$ point in the first BZ. This gap has an amplitude of ~0.3 eV and is centered at approximately −1.25 eV. However, these two intense bands are not observed at the $\bar{\Gamma }$ point in the first BZ. This phenomenon is attributed to the suppression of spectral weight at the $\bar{\Gamma }$ point of the first Brillouin zone—a behavior that has been reported in various systems and is believed to be related to the dipole selection rules for optical transitions in ARPES measurements, as well as band symmetry [42,43,44].

Figure 3c shows ARPES spectra of the *p*(2 × 2) superstructure along the $\bar{\Gamma }-\bar{K}-{\bar{M}}_{2}$ direction. The intensity of the Cu(111) substrate *sp* band remains prominent. The bands at the $\bar{\Gamma }$ point in Figure 3c are similar to those at the $\bar{\Gamma }$ point in Figure 3a. A conical feature is observed at the ${\bar{M}}_{2}$ point at roughly −1.8 eV (labeled as S_M2_ in Figure 3c), which looks like a Tamm state of Cu(111) [45,46,47].

Notably, the ~0.3 eV gap at ${\bar{\Gamma }}_{2}$ is a local feature between the hybrid Cu-Sn band (S_H_) and pure Cu *sp* band (S_L_), both of which lie below Fermi level (*E*_F_). The overall system remains metallic, as confirmed by ARPES (Cu *sp* band extending to *E*_F_).

### 3.4. DFT Verification of Electronic Structure and SOC Effects

**Figure 4:**
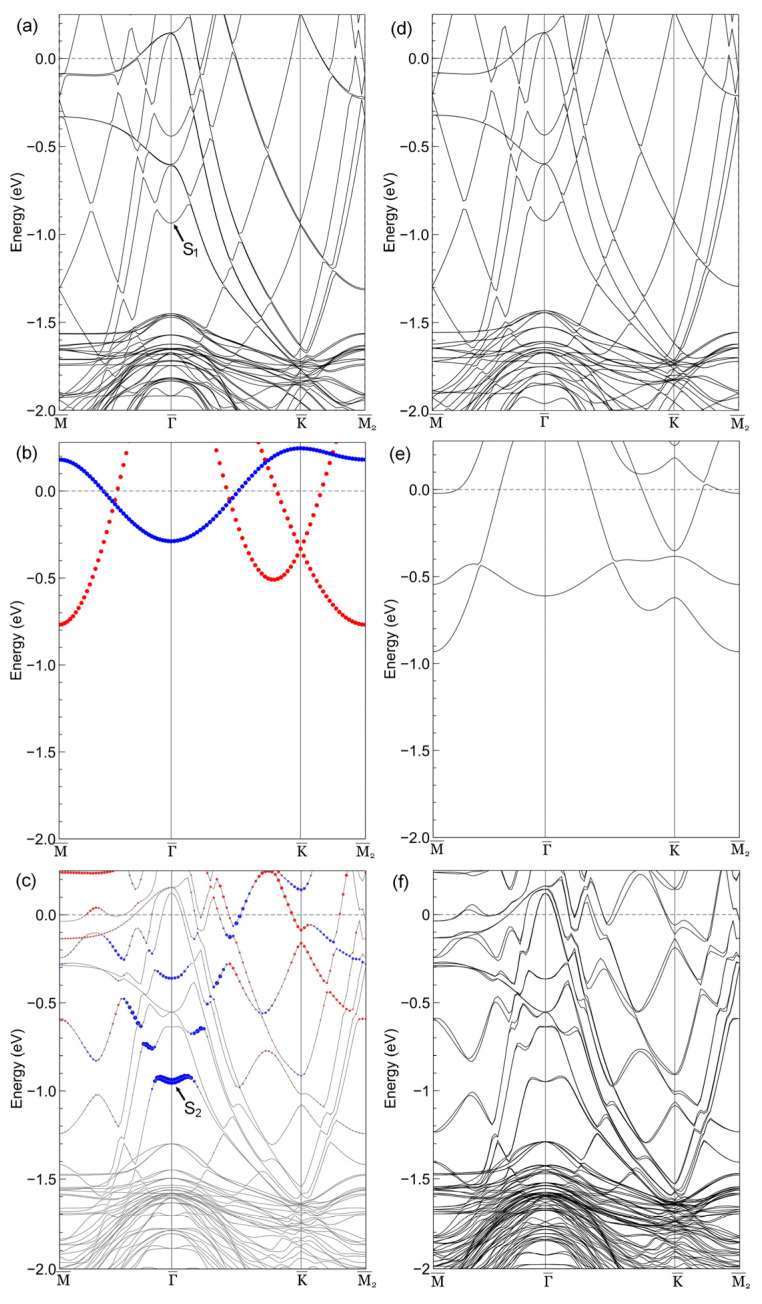
Band structures for clean Cu(111) (**a**,**d**), freestanding Sn film (**b**,**e**) and the *p*(2 × 2) Sn/Cu(111) superstructure (**c**,**f**) excluding (**a**–**c**) and including (**d**–**f**) the SOC. The size of red (blue) dots represents the weight of the contribution from the *p_xy_* (*p_z_*) orbital of Sn. Energy is referenced to the Fermi level (*E_F_* = 0).

To complement our analysis, we calculated the band structures of the clean Cu(111) surface, a freestanding single-layer Sn film, and the *p*(2 × 2) Sn/Cu(111) superstructure (presented in Figure 4a–c, respectively), with fatband projections of Sn atomic states. The corresponding band structures including SOC are shown in Figure 4d–f, respectively. Since SOC couples orbital angular momentum with spin momentum and mixes the *p_xy_* and *p_z_* orbitals of Sn atoms, the band structures including SOC were not projected onto the *p_xy_* and *p_z_* orbitals of Sn in Figure 4d–f. For intuitive comparison, a (2 × 2) supercell was used when calculating the band structure of the clean Cu(111) surface, so the band structure of the (1 × 1) structure was folded into that of the (2 × 2) structure (Figure 4a,d).

#### 3.4.1. Band Structure Decomposition and Sn-Cu Hybridization

A comparison between Figure 4b,c reveals that the *p_xy_* orbitals of Sn atoms (denoted as red dots in Figure 4c) undergo strong hybridization with substrate states, making them indistinguishable from the latter. In contrast, the *p_z_* orbitals of Sn atoms (denoted as blue dots in Figure 4c) remain predominantly unaffected and can be visualized via orbital projection (Figure 4c).

By comparing Figure 4a,c, we found that the adsorption of Sn atoms induces two major changes in the band structure. First, one of the Cu(111) bands originally located at ~−0.84 eV at the $\bar{\Gamma }$ point (labeled as S_1_ in Figure 4a) shifts downward to ~−0.96 eV (labeled as S_2_ in Figure 4c). The gap below the shifted band (S_2_ in Figure 4c) has an amplitude of ~0.34 eV, which is close to the experimental value. Furthermore, the bands at the $\bar{\Gamma }$ point become flatter than those of the clean Cu(111) surface, which is consistent with the experimental observation (Figure 3b). The downward shift in the S_1_ band and the flattening of the bands upon Sn adsorption primarily arise from the strong hybridization between the Sn orbitals and the surface electronic states of the Cu(111) substrate.

The DFT band structure confirms the metallic character of the *p*(2 × 2) superstructure: Sn *p_xy_* orbitals (red dots) hybridize with Cu *d*/*sp* bands to form states that cross *E*_F_, while the ~0.34 eV local gap between S_H_ and S_L_ bands does not disrupt metallicity.

#### 3.4.2. Spin–Orbit Coupling Effects

As a heavy element, Sn is expected to exhibit a strong SOC effect [48]. After including SOC, the band structure of the freestanding Sn film undergoes significant changes (Figure 4e). For example, a gap centered at ~−0.5 eV opens at the K point, with a magnitude of ~0.27 eV. In contrast, the SOC effect of Cu atoms is negligible: the splitting of the Cu(111) surface state induced by SOC is below 30 meV [49,50]. Thus, the band structures of Cu(111) above −1.5 eV appear nearly identical with and without SOC (Figure 4a,d). For the *p*(2 × 2) Sn/Cu(111) superstructure, the maximum band splitting above −1.5 eV (with SOC included) is ~75 meV, which is also relatively small.

The strong hybridization between the Sn adatoms and the Cu(111) substrate plays a critical role in moderating the pronounced SOC effects observed in freestanding Sn film. This hybridization leads to the mixing of Sn electronic states with those of the Cu substrate, resulting in wavefunctions that are more delocalized and less atomic-like in character. Since the atomic SOC strength is intrinsically linked to the localization and orbital character of the electronic states, this delocalization effectively dilutes the influence of Sn’s heavy-atom SOC. Consequently, while a freestanding Sn layer exhibits significant SOC-driven phenomena such as large band gaps, the hybridized interface in the *p*(2 × 2) superstructure shows only a modest SOC-induced band splitting.

#### 3.4.3. Substrate-Mediated Sn-Sn Coupling and Band Dispersion

The *p*(2 × 2) Sn/Cu(111) superstructure exhibits a Sn-Sn distance of ~5.1 Å (twice the lattice constant of the Cu(111) substrate, Section 3.1), which far exceeds the sum of Sn’s covalent (~1.41 Å) and vdW (~2.17 Å) radii—ruling out any significant direct Sn–Sn covalent or vdW interaction. Despite this long-range separation, the DFT band structure of the *p*(2 × 2) Sn/Cu(111) system (Figure 4c) clearly shows dispersion of Sn-derived bands (both *p_xy_* and *p_z_* orbitals, labeled by red and blue dots, respectively). This dispersion indicates non-negligible electronic coupling between spatially distant Sn atoms, which must be attributed to substrate mediation by the metallic Cu(111) surface [51].

To elucidate the underlying mechanism, it is necessary to distinguish the origin of this dispersion from that in the freestanding Sn film model (Figure 4b,e). In the freestanding case, the band dispersion arises from long-range hopping, while strong long-range Coulomb interactions govern its correlated electronic states [52,53]. In contrast, for the Sn/Cu(111) system, the dispersion implies non-negligible electronic coupling between spatially distant Sn atoms, which we attribute predominantly to an indirect, substrate-mediated mechanism [51].

In the *p*(2 × 2) Sn/Cu(111) superstructure, Sn adatoms are not electronically isolated; instead, their interactions are facilitated indirectly through the extended electronic states of the Cu(111) substrate. Each Sn adatom introduces a local perturbation to the Cu(111) surface potential, generating localized electronic states centered on the Sn site. When Sn atoms are arranged in the periodic *p*(2 × 2) lattice (Figure 2), these localized Sn states hybridize with the delocalized surface (e.g., Cu *sp* bands) and bulk (e.g., Cu *d* bands) electronic states of the Cu(111) substrate. The Cu substrate thus acts as an “electronic bridge,” enabling wavefunction overlap between Sn atoms over the ~5.1 Å distance: the electronic perturbation from one Sn atom propagates through the substrate’s delocalized bands to adjacent Sn atoms, leading to the formation of coherent, dispersive Sn-derived bands.

Notably, the hybridization in the *p*(2 × 2) Sn/Cu(111) system is predominantly vertical (i.e., between Sn orbitals and the Cu substrate), while the in-plane dispersion of Sn-derived bands emerges as a secondary property enabled by the substrate’s two-dimensional electron gas. This vertical hybridization explains the distinct behavior of Sn’s *p_z_* and *p_xy_* orbitals in the orbital-projected DFT band structure (Figure 4c): the Sn *p_z_* orbital—oriented perpendicular to the Cu(111) surface—undergoes weaker hybridization with the substrate, remaining relatively sharp and identifiable; in contrast, the Sn *p_xy_* orbitals—lying parallel to the surface—strongly hybridize with Cu *d*/*sp* bands, losing their distinct orbital identity and merging into the substrate’s band manifold.

This substrate-mediated coupling further rationalizes why Sn-derived bands follow the symmetry of the Cu(111) Brillouin zone (Figure 3a): their dispersion maxima and minima align with the same high-symmetry points ( $\bar{\Gamma }$, $\bar{M}$, $\bar{K}$) as the Cu substrate bands, confirming that the substrate’s electronic structure dictates the momentum-dependent behavior of Sn-derived states. Additional support for this picture comes from the spin–orbit coupling (SOC) effect: the maximum SOC-induced band splitting of Sn-derived bands in the *p*(2 × 2) system is ~75 meV (Figure 4f), which is significantly smaller than the splitting expected in systems with direct, strong Sn–Sn interactions. This small splitting confirms the absence of strong direct Sn–Sn bonding and reinforces the role of the Cu substrate in mediating inter-Sn electronic coupling.

#### 3.4.4. Origin of the Band Gap Discrepancy: Experimental and Theoretical Considerations

The minor discrepancy (~0.04 eV) between the DFT-calculated local gap (0.34 eV) and the ARPES measurement (~0.3 eV) stems from well-understood experimental and computational factors.

1. Nature of the Local Gap and Performance of PBE-GGA

It is essential to distinguish the nature of this gap. The ~0.3–0.34 eV feature is not a fundamental band gap but a local hybridization gap arising from the energy separation between the hybridized Sn-Cu band (S_H_) and the Cu substrate band (S_L_). Such local gaps are governed by short-range orbital hybridization, which semilocal functionals like PBE-GGA describe accurately, unlike the long-range electron correlations that control global gaps in semiconductors.

2. Experimental Broadening Lowers the Apparent Gap

It is critical to distinguish the intrinsic gap from the apparent gap measured by ARPES. The apparent gap measured by ARPES is reduced relative to the intrinsic gap, primarily due to instrumental broadening from the ~30 meV energy resolution and spectral convolution with the intense Cu(111) *sp* band. The finite resolution smears the band edges, while the intense Cu(111) *sp* band partially obscures the weaker Sn-derived S_H_ band, blurring the gap’s upper edge. Together, these effects reduce the apparent gap by ~0.03–0.04 eV, accounting for most of the observed discrepancy.

3. Computational Refinements Determine the Theoretical Gap

Our DFT setup is designed to recover the intrinsic gap, explaining the remaining offset:

k-point Sampling: The converged 28 × 28 × 1 k-mesh ensures precise sampling of the band edges.

Gaussian Smearing: Convergence tests confirm our choice of 0.1 eV smearing, necessary for SCF convergence in this metallic system, does not artificially reduce the gap (Supplementary Table S5).

Thus, the 0.04 eV difference is not a failure of the PBE functional but a natural consequence of comparing a smeared experimental measurement with a precise theoretical calculation of the intrinsic gap. The discrepancy falls within the combined uncertainties and does not undermine the excellent overall agreement.

#### 3.4.5. Reconciling ARPES Dispersion with DFT Band Structure: The Role of Band Unfolding

The apparent discrepancy between the parabolic dispersion observed by ARPES near the ${\bar{\Gamma }}_{2}$ point (Figure 3b) and the complex set of bands predicted by DFT at the $\bar{\Gamma }$ point (Figure 4c) can be resolved by considering the combined effects of Brillouin zone folding, substrate-adlayer hybridization, and photoemission selection rules. This analysis is essential for a direct comparison between supercell DFT calculations and ARPES measurements.

Our *p*(2 × 2) supercell DFT calculation folds the pristine Cu(111) surface Brillouin zone, causing multiple substrate bands—originally distributed across the (1 × 1) zone—to converge at the $\bar{\Gamma }$ point of the supercell Brillouin zone (Figure 4a). The introduction of Sn adatoms induces weak hybridization between these folded Cu states and Sn-derived orbitals, particularly the Sn *p_z_* orbital (denoted by blue dots in Figure 4c). This process generates the multitude of bands visible in Figure 4c, representing the full convolution of substrate and adlayer states at the supercell level.

A rigorous comparison with ARPES requires the concept of band unfolding [54,55]. The ARPES intensity often approximates the unfolded spectral function, where each eigenvalue from the supercell calculation is projected back into the original (1 × 1) Brillouin zone with a momentum-dependent spectral weight. The prominent parabolic dispersion observed experimentally at ${\bar{\Gamma }}_{2}$ corresponds to the branch of the hybridized Sn *p_z_*–Cu substrate complex that carries the highest spectral weight in the unfolded spectrum. The other DFT bands in this energy range, while physically present, possess negligible spectral weight at the ${\bar{\Gamma }}_{2}$ point or are suppressed by dipole selection rules (as noted in Section 3.3), rendering them invisible to ARPES.

Therefore, the ARPES signal represents a weighted projection of the hybridized electronic structure, with the parabolic feature being its most intense and experimentally detectable manifestation. This interpretation coherently unifies the DFT and ARPES results.

#### 3.4.6. Assessment of Van Der Waals Corrections

To ensure the robustness of our conclusions against the choice of functional, we investigated the effect of vdW interactions using the DFT-D3(BJ) scheme. The inclusion of D3(BJ) led to a modest increase (~7%) in the adsorption energy and a slight decrease (~2%) in the Sn-surface distance (Supplementary Table S6). These apparently contradictory trends—reduced binding strength with shorter bond length—align with recent critical assessments of D3 corrections in metallic systems [39], where such behavior is identified as a computational artifact arising from artificial overbinding and distortion of the potential energy surface.

Crucially, the relative stability of the adsorption sites remains unchanged by the D3(BJ) correction, and the electronic structure—particularly the local band gap at the $\bar{\Gamma }$ point—shows only minimal variation (~3% decrease). These findings reinforce that the chemical bonding in the *p*(2 × 2) Sn/Cu(111) system is dominated by strong metallic hybridization, which is adequately captured by the PBE functional. The excellent agreement between our PBE-calculated band structure and the ARPES measurements further supports this conclusion. Therefore, while vdW interactions were explicitly tested, the physical picture presented in this work, based on PBE, remains robust.

In summary, our ARPES measurements show good agreement with the proposed *p*(2 × 2) model when compared with the DFT-calculated band structure.

## 4. Conclusions

In this work, we investigated the geometry and electronic structure of Sn atoms adsorbed on a Cu(111) surface at low temperature using a combination of STM, LEED, ARPES measurements, and DFT simulations. By integrating LEED, STM observations, and DFT calculations, we found that Sn atoms adsorb at the three-fold *hcp* sites on the Cu(111) surface, with one Sn atom per *p*(2 × 2) unit cell. Furthermore, the DFT-calculated band structure shows good agreement with the ARPES measurements. Nevertheless, our *p*(2 × 2) Sn/Cu(111) structure (submonolayer, <0.25 ML) differs from the reported honeycomb-like or buckled stanene, highlighting that coverage are key parameters governing phase selection in the Sn/Cu(111) system. The diffusion and aggregation of adatoms are influenced by various factors such as substrate temperature, surface morphology, and adatom coverage. Additional research is needed to clarify the underlying mechanism of atomic arrangement in this system.

## Figures and Tables

**Table 1:** Adsorption energies (*∆E*_ad_) and Sn-surface distances (*d*_Sn-surf_) of the *p*(2 × 2) Sn/Cu(111) overlayer structures (ordered array of Sn atoms, one Sn atom per *p*(2 × 2) supercell) after structural optimization. All initial adsorption sites are retained post-relaxation. The most stable site for each composition is presented in bold.

Site	*∆E*_ad_ (eV)	*d*_Sn-surf_ (Å)
Top	−4.10	2.75
bridge	−4.15	2.56
*hcp*	**−4.1** **7**	**2.53**
*fcc*	−4.16	2.53

## Data Availability

Data are contained within the article.

## Supplementary Materials

The following supporting information can be downloaded at: https://www.mdpi.com/article/10.3390/nano15211684/s1. Table S1: Convergence test of total energy as a function of the wave function cutoff energy (charge density cutoff fixed at 10×) for the *p*(2 × 2) Sn/Cu(111) superstructure. Table S2: Convergence test of the total energy with respect to the Monkhorst-Pack k-point mesh for the *p*(2 × 2) Sn/Cu(111) superstructure. Table S3: Convergence test of the adsorption energy (Δ*E*_ad_) and Sn-surface distance (*d*_Sn-surf_) for Sn at the *hcp* site as a function of vacuum thickness in the *p*(2 × 2) Sn/Cu(111) superstructure. Table S4: Convergence test of the adsorption energy (Δ*E*_ad_) and Sn-surface distance (*d*_Sn-surf_) for Sn at the *hcp* site as a function of the number of Cu layers in the *p*(2 × 2) Sn/Cu(111) superstructure. Table S5: Convergence test of the total energy, adsorption energy (Δ*E*_ad_) for Sn at the *hcp* site, and local gap energy between the band S_H_ and S_L_ at $\bar{\Gamma }$ point as a function of smearing width in the *p*(2 × 2) Sn/Cu(111) superstructure. Table S6: Comparison of GGA-PBE and GGA-PBE-D3(BJ) predictions for key properties of the Sn/Cu(111) system: adsorption energy (Δ*E*_ad_) at the *hcp* site, Sn-surface distance (*d*_Sn-surf_), and the local S_H_-S_L_ band gap at the $\bar{\Gamma }$ point.
