# Supplementary material for: Distinct 2D p(2 × 2) Sn/Cu(111) Superstructure at Low Temperature: Experimental Characterization and DFT Calculations of Its Geometry and Electronic Structure
Source: Nanomaterials (Basel). 2025 Nov 6;15(21):1684. doi: 10.3390/nano15211684 (PMC12610681; doi:10.3390/nano15211684)
Supplement: Supplementary file 1 [file nanomaterials-15-01684-s001.zip › nanomaterials-3931301-supplementary.pdf]

Distinct 2D $p(2 \times 2)$ Sn/Cu(111) Superstructure at Low Temperature: Experimental Characterization and DFT Calculations of Its Geometry and Electronic Structure

Xihui Liang [1,2,*], Dah-An Luh [2,3,*] and Cheng-Maw Cheng [3]

[1] School of Arts and Sciences, Guangzhou Maritime University, Guangzhou 510725, China
[2] Department of Physics, National Central University, Taoyuan 320317, Taiwan, China
3 National Synchrotron Radiation Research Center, Hsinchu 300092, Taiwan, China; makalu@nsrrc.org.tw (C.-M.C.)
* Correspondence: liangxihui@gzmtu.edu.cn (X.L.); luh.dah.an@gmail.com (D.-A.L.)

Supplementary Table S1 Convergence test of total energy as a function of the wave function cutoff energy (charge density cutoff fixed at 10×) for the $p(2 \times 2)$ Sn/Cu(111) superstructure..

Wave Function Cutoff (eV)	Charge Density Cutoff (eV)	Total Energy (eV/atom)	Relative Change (meV/atom)
600	6000	-16.3350	-
700	7000	-16.3352	0.2
800	8000	-16.3353	0.1
900	9000	-16.3353	0.0

Supplementary Table S2 Convergence test of the total energy with respect to the Monkhorst-Pack k-point mesh for the $p(2\times2)$ Sn/Cu(111) superstructure.

k-Mesh	Total Energy (eV/atom)	ΔE_{ad} (*hcp*, eV)	Local Gap Energy (eV)
16×16×1	-16.334	4.17	0.33
20×20×1	-16.335	4.17	0.34
24×24×1	-16.335	4.17	0.34
28×28×1	-16.335	4.17	0.34
32×32×1	-16.335	4.17	0.34

Supplementary Table S3 Convergence test of the adsorption energy (ΔE_{ad}) and Sn-surface distance ($d_{Sn\text{-}surf}$) for Sn at the hcp site as a function of vacuum thickness in the *p*(2×2) Sn/Cu(111) superstructure.

**Vacuum Thickness (Å)**	**ΔE_{ad} (eV)**	**$d_{Sn\text{-}Surf}$ (Å)**
10	-4.16	2.53
12	-4.17	2.53
15	-4.17	2.53
18	-4.17	2.53

Supplementary Table S4 Convergence test of the adsorption energy (ΔE_{ad}) and Sn-surface distance ($d_{Sn\text{-}surf}$) for Sn at the hcp site as a function of the number of Cu layers in the *p*(2×2) Sn/Cu(111) superstructure.

**Number of Cu Layers**	**ΔE_{ad} (eV)**	**$d_{Sn\text{-}Surf}$ (Å)**
4	-4.14	2.54
5	-4.15	2.53
6	-4.17	2.53
7	-4.18	2.53

Supplementary Table S5 Convergence test of the total energy, adsorption energy (ΔE_{ad}) for Sn at the hcp site, and local gap energy between the band S_H and S_L at $\bar{\Gamma}$ point as a function of smearing width in the *p*(2×2) Sn/Cu(111) superstructure.

**Smearing width (eV)**	**Total Energy (eV/atom)**	**ΔE_{ad} (*hcp*, eV)**	**Local Gap Energy (eV)**
0.20	-16.335	4.16	0.33
0.15	-16.335	4.17	0.34
0.10	-16.335	4.17	0.34
0.05	-16.335	4.17	0.34

Supplementary Table S6 Comparison of GGA-PBE and GGA-PBE-D3(BJ) predictions for key properties of the Sn/Cu(111) system: adsorption energy (ΔE_{ad}) at the hcp site, Sn-surface distance ($d_{Sn\text{-}surf}$), and the local S_H-S_L band gap at the $\bar{\Gamma}$ point.

Parameter	GGA-PBE	GGA-PBE-D3(BJ)	Relative Change
Adsorption energy (ΔE_{ad}, eV) for hcp site	4.17	3.88	~7% increase (less negative)
Sn-substrate distance ($d_{Sn\text{-}surf}$) for hcp site	2.53	2.47	~2% decrease
Band gap between band S_H and S_L at $\bar{\Gamma}$ point (eV)	0.34	0.33	~3% decrease
